# A174 TESTING FOR AND INCIDENCE OF CELIAC DISEASE AUTOIMMUNITY SAW SIGNIFICANT BUT TEMPORARY DECLINES DURING COVID-19 PANDEMIC

**DOI:** 10.1093/jcag/gwad061.174

**Published:** 2024-02-14

**Authors:** J A King, S Coward, G G Kaplan, T Williamson

**Affiliations:** University of Calgary, Calgary, AB, Canada; University of Calgary, Calgary, AB, Canada; University of Calgary, Calgary, AB, Canada; University of Calgary, Calgary, AB, Canada

## Abstract

**Background:**

Celiac disease (CD) is an autoimmune disorder with various complications and long-term health risks. The COVID-19 pandemic affected the entire healthcare system—including screening for and diagnosing non-infectious conditions.

**Aims:**

Determine how the COVID-19 pandemic affected the testing for, and incidence of, CD autoimmunity.

**Methods:**

Using the population-based Alberta Medical Laboratory database, all tissue transglutaminase antibody tests (tTG-IgA) were identified in Alberta (April 2012 to March 2023). Incident cases of CD autoimmunity comprised of individuals newly positive for tTG-IgA between April 2015 and March 2023. Prevalent cases of CD were excluded based on previous tTG-IgA positivity and/or CD diagnosis codes in outpatient or inpatient settings. Average monthly percent changes and inflection points for testing and incidence rates were estimated using Joinpoint regression. Autoregressive integrated moving average models were performed to forecast rates if the pandemic had not occurred. Incidence rate ratios (IRRs) were estimated to compare a pre-pandemic era (April 2017 to March 2020) and a pandemic era (April 2020 to March 2023) overall and across subgroups.

**Results:**

In the pandemic era, 311,971 tTG-IgA tests were performed on 284,882 unique individuals (21.1 per 1000 person-years (Table 1)). Testing decreased by 23.4% per month from February 2020 until May 2020 (Figure 1A). From June 2020 to August 2020, testing rates increased by 22.3% per month and then remained stable until the end of the study period. There were 4907 new cases of CD autoimmunity in the pandemic era, with an incidence of 36.3 per 100,000 person-years (Table 1). Incidence rates decreased by 14.1% per month from January 2020 until April 2020 (Figure 1B). Subsequently, incidence continued to rise by 2.0% per month from May 2020 until the end of the study period.

**Conclusions:**

The COVID-19 pandemic appears to have had a minor impact on identifying potential CD. Future surveillance is needed to determine if the pandemic affected the diagnostic pathway to biopsy-confirmed CD through upper endoscopy procedures and any adverse outcomes individuals may have had from delayed diagnosis.

Table 1: Testing and incidence rates for CD autoimmunity in Alberta in pre-pandemic era (April 2017 to March 2020) and pandemic era (April 2020 to March 2023)

IRR=incidence rate ratio; CI=confidence interval

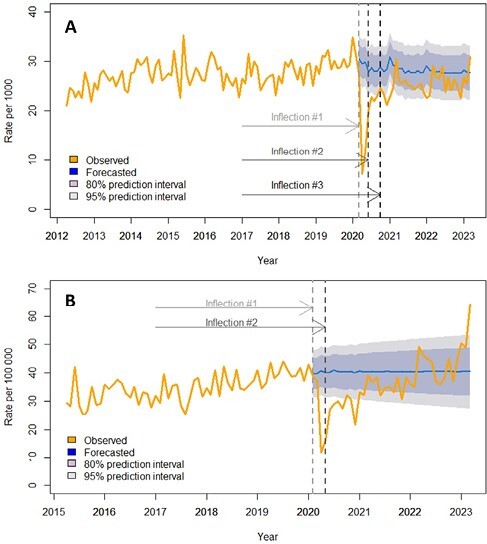

**Figure 1:** Testing (A) and incidence (B) rates for CD autoimmunity before and after the start of the COVID-19 pandemic

**Funding Agencies:**

CAG, CIHRhttps://triangleprogram.org

**Table:** 

Category	Testing per 1,000 person-years			Incidence per 100,000 person-years		
	Pre-pandemic	Pandemic	IRR (95% CI)	Pre-pandemic	Pandemic	IRR (95% CI)
Overall	24.0	21.1	0.88 (0.87-0.88)	37.1	36.3	0.98 (0.94-1.02)
Children (0-17 yrs)	25.0	23.9	0.96 (0.95-0.97)	50.4	55.0	1.09 (1.02-1.17)
Adults (18 to 64 yrs)	24.6	20.9	0.85 (0.84-0.86)	36.4	34.1	0.94 (0.89-0.99)
Seniors (65+ yrs)	20.3	18.0	0.89 (0.87-0.90)	18.1	17.8	0.96 (0.85-1.15)
Females	29.8	26.5	0.89 (0.88-0.89)	47.6	46.4	0.98 (0.93-1.02)
Males	18.3	15.8	0.86 (0.85-0.87)	26.7	26.3	0.98 (0.92-1.05)
Metropolitan	25.6	22.4	0.87 (0.86-0.87)	39.4	38.1	0.97 (0.92-1.01)
Non-metropolitan	19.8	17.7	0.89 (0.88-0.90)	31.3	31.6	1.01 (0.93-1.09)

